# Ultrasonographic Prenatal Diagnosis: Unveiling the Path to Improved Antenatal Care

**DOI:** 10.3390/jcm12134450

**Published:** 2023-07-03

**Authors:** Roberta Granese, Ferdinando Antonio Gulino, Giosuè Giordano Incognito, Stefano Cianci, Canio Martinelli, Alfredo Ercoli

**Affiliations:** 1 Unit of Gynecology and Obstetric, Department of Biomedical and Dental Sciences and Morpho Functional Imaging, University Hospital “G. Martino” of Messina, 98100 Messina, Italy; 2 Unit of Gynecology and Obstetric, Department of Human Pathology of Adults and Developmental Age, University Hospital “G. Martino” of Messina, 98100 Messina, Italy; docferdi@hotmail.it (F.A.G.); stefanoc85@hotmail.it (S.C.); caniomartinelli.md@gmail.com (C.M.); alfercoli@yahoo.it (A.E.); 3 Department of General Surgery and Medical Surgical Specialties, University of Catania, 95125 Catania, Italy; giordanoincognito@gmail.com

The realm of prenatal diagnosis has witnessed remarkable advancements in recent years, primarily due to the widespread use of ultrasonography. This non-invasive imaging technique has revolutionized the way we perceive and manage pregnancies, allowing us to explore the intricate details of fetal development with unprecedented clarity [1]. Ultrasonographic prenatal diagnosis has emerged as an invaluable tool for identifying potential abnormalities and facilitating timely interventions, leading to improved antenatal care and better outcomes for both mother and child. Ultrasonographic prenatal diagnoses provide an intimate window to the womb, allowing healthcare professionals to observe and evaluate various aspects of fetal development. From the early stages of pregnancy, ultrasound scans enable the detection of multiple gestations, determination of gestational age, assessment of embryonic viability, and evaluation of the location of the pregnancy [2,3]. As the pregnancy progresses, detailed imaging can unveil the growth and formation of vital organs, skeletal structures, and the intricate network of blood vessels within the developing fetus. One of the most significant contributions of ultrasonographic prenatal diagnosis is its ability to detect congenital anomalies at an early stage. Through meticulous examination, anomalies such as neural tube defects, heart malformations, abdominal wall defects, and skeletal abnormalities can be identified, allowing healthcare providers to prepare for potential interventions and coordinate appropriate medical care [4,5,6,7,8,9,10]. This proactive approach aids in optimizing treatment plans, minimizing postnatal complications, and providing crucial support to parents in understanding and planning for their child’s healthcare needs. In addition to anomaly detection, ultrasonographic prenatal diagnosis plays a pivotal role in assessing fetal well-being throughout pregnancy. It enables the evaluation of amniotic fluid volume, placental health, and fetal growth patterns, allowing healthcare providers to monitor and manage conditions such as intrauterine growth restriction, placental insufficiency, and oligohydramnios [11,12,13]. Regular ultrasound examinations reassure expectant parents, identifying and managing potential issues promptly [14]. In certain cases, when further investigation or treatment is required, ultrasonographic prenatal diagnosis serves as a guiding tool for invasive procedures. Procedures such as chorionic villus sampling (CVS), amniocentesis, and fetal blood sampling can be performed under real-time ultrasound guidance, thereby minimizing the risk of complications and improving the accuracy of the procedure [15]. This level of precision enhances the safety and efficacy of these interventions, providing healthcare professionals with the necessary information for appropriate decision-making [16]. Ultrasonographic prenatal diagnosis has revolutionized the field of antenatal care, empowering healthcare providers to better understand, detect, and manage fetal anomalies and complications. The ability to visualize the developing fetus in utero has transformed the experience of pregnancy for both parents and medical professionals. Ultrasonography has become indispensable by enabling early detection, facilitating interventions, and improving overall prenatal care, leading to enhanced outcomes and fostering a sense of confidence and well-being in expectant families. As technology continues to evolve, we anticipate further advancements that will refine and expand the capabilities of ultrasonographic prenatal diagnosis, bringing us closer to a future in which every pregnancy receives the best possible care.
